# Comparable Rates of Integrated Myofibrillar Protein Synthesis Between Endurance-Trained Master Athletes and Untrained Older Individuals

**DOI:** 10.3389/fphys.2019.01084

**Published:** 2019-08-30

**Authors:** James McKendry, Brandon J. Shad, Benoit Smeuninx, Sara Y. Oikawa, Gareth Wallis, Carolyn Greig, Stuart M. Phillips, Leigh Breen

**Affiliations:** ^1^School of Sport, Exercise and Rehabilitation Sciences, University of Birmingham, Birmingham, United Kingdom; ^2^Department of Kinesiology, McMaster University, Hamilton, ON, Canada; ^3^NIHR Birmingham Biomedical Research Centre, Birmingham, United Kingdom; ^4^MRC-Arthritis Research UK Centre for Musculoskeletal Ageing Research, University of Birmingham, Birmingham, United Kingdom

**Keywords:** muscle, anabolism, master athlete, resistance exercise, sarcopenia

## Abstract

**Background:**

An impaired muscle anabolic response to exercise and protein nutrition is thought to underpin age-related muscle loss, which may be exacerbated by aspects of biological aging that may not be present in older individuals who have undertaken long-term high-level exercise training, or master athletes (MA). The aim of this study was to compare rested-state and exercise-induced rates of integrated myofibrillar protein synthesis (iMyoPS) and intracellular signaling in endurance trained MA and healthy age-matched untrained individuals (Older Controls).

**Methods:**

In a parallel study design, iMyoPS rates were determined over 48 h in the rested-state and following a bout of unaccustomed resistance exercise (RE) in OC (*n* = 8 males; 73.5 ± 3.3 years) and endurance-trained MA (*n* = 7 males; 68.9 ± 5.7 years). Intramuscular anabolic signaling was also determined. During the iMyoPS measurement period, physical activity was monitored via accelerometry and dietary intake was controlled.

**Results:**

Anthropometrics, habitual activity, and dietary intake were similar between groups. There was no difference in rested-state rates of iMyoPS between OC (1.47 ± 0.06%⋅day^–1^) and MA (1.46 ± 0.08%⋅day^–1^). RE significantly increased iMyoPS above rest in both OC (1.60 ± 0.08%⋅day^–1^, *P* < 0.01) and MA (1.61 ± 0.08%⋅day^–1^, *P* < 0.01), with no difference between groups. Akt^*Thr*308^ phosphorylation increased at 1 h post-RE in OC (*P* < 0.05), but not MA. No other between-group differences in intramuscular signaling were apparent at any time-point.

**Conclusion:**

While our sample size is limited, these data suggest that rested-state and RE-induced iMyoPS are indistinguishable between MA and OC. Importantly, the OC retain a capacity for RE-induced stimulation of skeletal muscle remodeling.

## Introduction

The rapidly expanding aging population presents a substantial concern amongst healthcare professionals and researchers alike. By the year 2050, ∼23 million people will be aged 60 years or older, accounting for >30% of the UK population (Office for National Statistics)^1^. The overwhelming challenge is that the time spent in good health in older age (i.e., health-span) is not expanding at the same rate as average lifespan (Seals et al., 2016). Thus, it is critical that effective strategies to minimize the gap between life- and health-span are identified. Skeletal muscle is vital for the maintenance of physical function, nutrient deposition and basal metabolism (Frontera and Ochala, 2015). Aging leads to a progressive loss of skeletal muscle [termed “sarcopenia” (Rosenberg, 1989)], which progresses at a rate of 0.5–1% per annum from the 5th decade, alongside 3–5-fold greater reductions in strength (Janssen et al., 2000; Delmonico et al., 2009). Accordingly, sarcopenia may drive the development, and progression, of many adverse age-related health events (Narici and Maffulli, 2010; Biolo et al., 2014) and force a dependence on external healthcare services (Sonn, 1996; Pinedo-Villanueva et al., 2019).

Sarcopenia progression is thought to be underpinned by inherent aging factors (i.e., hormonal changes) and aggravated by environmental and lifestyle factors (i.e., poor nutrition, obesity, and reduced activity) (Narici and Maffulli, 2010; Smeuninx et al., 2017), that blunt the muscle protein synthesis (MPS) response to normally robust anabolic stimuli, such as hyperaminoacidemia and resistance exercise (RE; Breen and Phillips, 2011; Shad et al., 2016). This age-related muscle “anabolic resistance” may be underpinned by impairments in translational efficiency in the mechanistic target of rapamycin complex 1 (mTORC1) signaling pathway (Kumar et al., 2009; Brook et al., 2016). Whilst RE is effective at enhancing muscle anabolic sensitivity (Timmerman et al., 2012; Yang et al., 2012) and augmenting muscle mass and strength in older individuals (Frontera et al., 1988; Fiatarone et al., 1990), myofibrillar adaptive remodeling responses are attenuated compared with younger individuals (Kumar et al., 2009; Brook et al., 2016; Phillips et al., 2017). However, chronic structured exercise training is known to alter acute muscle protein turnover rates in young and older individuals (Yarasheski et al., 1999; Short et al., 2004; Wilkinson et al., 2008). Therefore, commencing exercise training in early adulthood, and continuing this practice through middle-to-older age, may offset or delay the onset of muscle anabolic resistance, with implications for age-related muscle loss.

Highly active older individuals who have maintained structured exercise training habits [Master Athletes, (MA)] display superior indices of physiological function (VO_2max_ and strength), muscle morphology and, typically, a more favorable body composition than their untrained age-matched counterparts (McKendry et al., 2018, 2019), which could potentially be explained by differences in MPS. The only study to date to investigate MPS in MA, reported that highly trained master triathletes (>50 years) displayed lower MPS rates following a bout of downhill running than younger triathletes (Doering et al., 2016). However, rested-state MPS was not measured, preventing firm conclusions regarding the net MPS response to the exercise stimulus. Furthermore, a comparison between MA and age-matched untrained older individuals is required to understand how long-term exercise training modulates MPS. It has been shown that physiological function (i.e., VO_2max,_ strength) and indices of muscle morphology appear to deteriorate at a similar relative rate in highly trained MA and untrained older individuals (Pearson et al., 2002; Pollock et al., 2015, 2018). However, the impact of chronic exercise training on *in vivo* metabolic and molecular regulation of skeletal muscle mass in older age, is yet to be resolved. Given the influence of physical activity and chronic training on muscle protein turnover, it is possible that the acute (i.e., hours-to days) muscle remodeling response to exercise may differ between long-term trained MA than untrained age-matched individuals.

The aim of the present study was to compare 48 h rested-state and RE-induced integrated myofibrillar protein synthesis (iMyoPS) rates between MA and age-matched untrained individuals and to establish the acute intramuscular signaling response to an acute bout of RE contraction. We hypothesized that rested-state iMyoPS rates would be similar between groups, but that RE-induced iMyoPS rates and intramuscular signaling responses would be greater in MA vs. age-matched untrained individuals, indicative of a greater (or maintained) capacity for muscle remodeling in long-term exercisers.

## Materials and Methods

### Participants

Eight untrained older male controls and 7 male master endurance athletes were recruited through local advertisement at athletics and cycling clubs, the British Masters Athletics Federation and the League of Veteran Racing Cyclists. Older control participants (60–80 years) were deemed eligible for the study only if they had maintained habitual activity levels and had not previously participated in any form of structured exercise training outside of recreational activities. Master athletes (60–80 years) were included only if they self-reported maintaining continuous endurance training at least twice per week for ≥20 years preceding the study. Participant anthropometric and training characteristics are detailed in Table 1. In our recent work, we provided a comprehensive characterization of physiological function and muscle morphology in a larger sample of MA and OC, distinct from the volunteers recruited for the current study (McKendry et al., 2019). Thus, we chose not to repeat these measurements in the present study due to the required time commitment and instead focused on *in vivo* measurement of iMyoPS and intracellular signaling mechanisms. All participants were informed of the purpose and methodology of the study, were deemed healthy by completion of a general health questionnaire assessment, and provided their written informed consent. Ethical approval was obtained through the East Midlands – Derby Research Ethics Committee (18/EM/0004) and conformed to the requirements of Research Governance at the University of Birmingham Research Governance, as the study sponsor.

**TABLE 1:** Participant anthropometric characteristics and training background.

	**OC (*N* = 8)**	**MA (*N* = 7)**	***P*-value**
Age (years)	73.5 ± 3.3	68.9 ± 5.7	0.071
Height (m)	1.73 ± 0.06	1.76 ± 0.08	0.362
Body mass (kg)	73.8 ± 8.5	70.0 ± 4.5	0.131
BMI (kg.m^–2^)	24.8 ± 3.3	22.0 ± 1.6	0.059
Whole-body LM (kg)	52.9 ± 3.6	52.0 ± 3.8	0.628
Whole-body FM (kg)	20.9 ± 6.9	16.0 ± 1.5	0.092
Body fat (%)	27.8 ± 6.7	23.6 ± 1.9	0.134
Skeletal muscle index (%)	72.2 ± 6.7	76.4 ± 1.9	0.140
Systolic blood pressure (mmHg)	134 ± 10	130 ± 8	0.430
Diastolic blood pressure (mmHg)	78 ± 9	81 ± 3	0.478
Training experience (years)	−	47.7 ± 14.8	−
Training frequency (sessions.week^–1^)	−	4.3 ± 1.6	−
Training duration (hrs.week^–1^)	−	8.4 ± 6.6	−
Training distance (km.week^–1^)	−	180 ± 29/55 ± 15	−

*Data presented as mean ± SD. LM, lean mass; FFM, fat-free mass; FM, fat mass. Training distance is separated into cyclists (*n* = 3, left) and runners (*n* = 4, right).*

### Study Design

**FIGURE 1:**
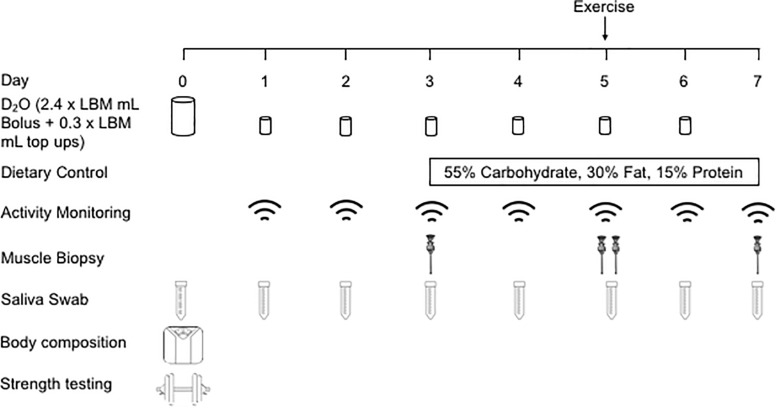
Schematic overview of the experimental design. LBM, lean body mass.

In a parallel study design, OC and MA were recruited to investigate the effects that chronic endurance training elicits on the regulation of muscle mass in an older population (detailed in the study schematic, Figure 1). Following initial study screening and consenting, participants reported to the School of Sport, Exercise and Rehabilitation Sciences (SportExR) laboratory on four separate occasions. For each visit, participants reported to SportExR in an overnight fasted-state and were asked to refrain from caffeine consumption on the day of the trial. Further, participants were asked to refrain from strenuous physical activity and alcohol for the duration of study involvement.

#### Visit 1

During the initial visit, participants underwent assessments of anthropometrics, body composition and provided a single saliva sample which was collected daily throughout study involvement. After a baseline assessment of blood pressure and body composition, participants underwent bilateral 1 repetition maximum (1RM) strength testing and familiarization to the exercise protocol. Following this, participants were given a bolus of the deuterated water (D_2_O) and daily top-ups for measurement of myofibrillar protein synthetic rates. Participants were supplied with a pedometer to monitor their habitual daily activity.

#### Visit 2

Seventy-two hours following visit 1, participants provided a single saliva sample and a muscle biopsy was collected. Participants were provided with a weight maintenance food parcel, matched for total calories and macronutrient content for the subsequent 4 days to standardize dietary conditions and reduce the influence of dietary variances between individuals.

#### Visit 3

Forty-eight hours following visit 2, participants provided a single saliva sample and a muscle biopsy was collected. Participants then completed a bout of leg RE, which involved performing 6 sets of 10 repetitions of leg extension exercise at 75% of their predetermined 1RM. Each set was separated by 2 min of passive rest. Participants then rested for 1 h before another muscle biopsy was collected to examine the acute intramuscular signaling response to the RE bout. RE was selected as this exercise mode delivered a targeted contractile stimulus that MA and OC were similarly unaccustomed to. This point is important as long-term exercise training modifies the protein synthetic response of functional protein fractions, with a shift toward exercise phenotype-specific responses. As such, there is potential for a preferential synthesis of mitochondrial proteins following aerobic exercise in endurance trained MA (Wilkinson et al., 2008). Furthermore, acute RE is a potent stimulus for myofibrillar protein remodeling and muscle maintenance in older individuals (Phillips et al., 2017).

#### Visit 4

Forty-eight hours following visit 3, participants reported to SportExR to provide a final saliva and muscle biopsy sample, after which involvement in the study was completed and participants were allowed to leave.

### Experimental Procedures

#### Body Mass, Height, and Composition

Body mass was determined by weighing each participant in loose clothing, without shoes, to the nearest 0.1 kg using a digital balance scale (Seca 813, Hamburg, Germany). Height measurements were made to the nearest 0.1 cm using a stadiometer (Seca 217, Hamburg, Germany). Participants underwent bioelectrical impedance analysis in order to determine fat and lean mass (Bodystat QuadScan 4000, Bodystat Ltd., Isle of Man, British Isles). Individuals were instructed to lay in a supine position for ∼10 min to allow for any fluid shifts and electrodes were attached to the right-side hand and foot. Skeletal muscle index was calculated as lean mass as a percentage of whole-body mass.

#### Blood Pressure

Blood pressure was measured using a standard fully automatic blood pressure monitor (OMRON M2, OMRON Healthcare UK Ltd., United Kingdom). Participants were asked to remove any clothing that obstructed the blood pressure cuff. Participants were seated with their legs uncrossed and back supported, encouraged to relax and refrain from talking during the assessment. This test was repeated three times and the lowest reading taken.

#### Leg Extension 1RM and Exercise Familiarization

Individuals underwent 1RM bilateral knee-extension strength testing (Elite Series – Leg Extension/Curl, Fitness Warehouse Ltd., Preston, Lancashire, United Kingdom). Participants were instructed on proper lifting technique and carried out a warm up set of 10 repetitions at ∼ 40% 1RM. Following which, the weight was increased and the repetitions reduced utilizing a standardized 1RM testing protocol adapted from Mayhew et al. (1992) until the participants could no longer lift the desired load, with correct form, for 1 repetition. Individuals provided feedback on the difficulty of each set using an adapted Borg Scale (CR-10) (Day et al., 2004; Buckley and Borg, 2011). Participants rested for 2 min between each set, and 3 min rest was provided between 1RM attempts.

#### Physical Activity and Dietary Control

Participants were provided with a standard, wrist-worn, pedometer (ID115HR LETSCOM, Hong Kong) to record the average total daily step count over the duration of the study. Participants were asked to refrain from strenuous exercise for the duration of study involvement (i.e., 7 days, see Figure 1). As such, step count monitoring during the study did not include habitual endurance exercise levels. Participants were provided with a standardized, weight-maintenance diet (∼60% carbohydrate, ∼23% fat and ∼17% protein) to consume during the study. Energy requirements were calculated using the Harris–Benedict equation (Harris and Benedict, 1919) multiplied by an activity factor of 1.35. Participants could choose from a selection of food options, including several pre-packaged microwavable options for lunch and dinner, pre-weighed breakfast ingredients (e.g., porridge oats, milk, and yogurt) and pre-packaged mid-meal snacks (snack bars, nuts). Water was consumed *ad libitum* and pre-weighed beverages (fruit juices and tea) were provided. Food was provided to participants between visit 2 and visit 4, for consumption over the course of iMyoPS measurement. Participants also consumed a beverage containing 20 g of whey isolate protein (MyProtein, Northwich, United Kingdom), following the second biopsy on visit 3 (i.e., 1 h post-exercise) to augment exercise-induced MPS similarly in MA and OC in the initial several hours of recovery (Yang et al., 2012).

#### Isotope Tracer Protocol

Stable isotopically labeled D_2_O was provided to participants throughout study involvement. To rapidly increase the body water enrichment of deuterium (^2^H) to ∼0.2%, participants consumed a bolus dose of D_2_O on day 1 (2.4 mL⋅kg lean body^–1^) mass (LBM). To maintain the ∼0.2% enrichment of body water throughout study involvement, participants consumed a daily top-up dose of D_2_O (0.3 mL⋅kg LBM^–1^). The D_2_O dosing relative to LBM was selected to achieve stable enrichment in the body water pool, which is predominantly confined to bone- and fat-free (i.e., lean) mass. Total body water enrichment was used as a surrogate for deuterated-alanine labeling as previously described (Wilkinson et al., 2014). All doses were consumed in the morning, immediately following saliva sample provision. Saliva samples were collected in the morning, immediately on waking. Participants chewed a cotton swab for ∼2 min or until completely saturated. Samples were stored in the fridge and returned during the next lab visit. Saliva was pressed out of the swab using a 5 mL syringe into two separate glass vials, sealed and stored at −20°C until later analysis.

#### Muscle Biopsy

Muscle biopsy samples were obtained from the quadriceps vastus lateralis under local anesthesia (1% lidocaine) using the Bergström needle technique (Bergstrom, 1975). Muscle biopsy tissue was quickly rinsed in ice-cold saline and blotted to remove any visible fat and connective tissue before being frozen in liquid nitrogen.

### Data Analyses

#### Myofibrillar Protein Synthesis

Muscle samples (∼30–35 mg) were homogenized (TissueLyser, Hilden, Germany) in a 2 mL Eppendorf in 500 μl ice cold homogenization buffer (25 mM Tris buffer [Tris–HCl, Trizma Base, 25 ml of Milli-Q H_2_O, pH 7.2], 1 PhosSTOP.

Tablet (Roche, Switzerland), 100 μl TritonX-100, 1 complete (Roche) mini protease inhibitor tab). Samples were centrifuged at 4500 rpm for 10 min at 4°C to separate the sarcoplasmic (supernatant) and myofibrillar fractions. The myofibrillar fraction was purified by adding 500 μl of DDH_2_0, vortexing for 5 s and centrifuging at 1500 rpm for 10 min at 4°C. Then, 1 ml of 0.3 M NaOH was added to the sample and vortexed for 5 s before being placed in a heating block at 50°C for 30 min (vortex 5 s every 10 min). Samples were then centrifuged at 10,000 rpm for 10 min at 4°C before the supernatant (containing the myofibrillar fraction) was removed and placed in a 4 ml glass screw-top tube. Next, 1 mL of 1 M perchloric acid was added to the tubes and centrifuged at 2500 rpm for 10 min at 4°C. After removing he supernatant, the myofibrillar protein pellet was washed in twice in 1 mL of 70% ethanol (centrifuging at 2500 rpm for 10 min at 4°C). Amino acids were liberated by adding 1 mL of Dowex resin (50WX8-200 resin; Sigma-Aldrich) and 1 ml of 1 M HCL before heating at 110°C for 72 h. The free amino acids were further purified on cation-exchange columns, dried and reconstituted in 0.1 M HCl before analysis by gas chromatography combustion isotope ratio mass spectrometry (Metabolic Solutions, Nashua, NH, United States). Muscle preparations were analyzed for deuterated-alanine (^2^H-alanine) with a Thermo Finnigan Delta V isotope ratio mass spectrometry coupled to a Thermo Trace GC Ultra with a gas chromatography combustion interface III and Conflow IV. The N-acetyl n-propyl ester of alanine was analyzed using a splitless injection and a Zebron ZB-5 column of 30 m × 0.25 mm × 0.50 μm film thickness (Phenomenex, Torrance, CA, United States). The gas chromatography oven was programed with an initial column temperature of 80°C with a 2-min hold, followed by a ramp of 30°C min^–1^ to 330°C. Eluents were directed into the pyrolysis reactor, heated at 1450°C, and converted to hydrogen gas (Metabolic Solutions, Nashua, NH, United States); as described previously (Bell et al., 2015). The injections were performed in duplicate all with a CV of less than 5%. The equipment underwent daily and intra-run calibration to ensure stability using an external standard. Saliva samples were analyzed for ^2^H enrichment by cavity ring-down spectroscopy (L2130-i, Picarro Inc., Santa Clara, CA, United States). The water phase of the saliva was injected six times, and the average of the last three measurements was used for data analysis. The ^2^H isotopic enrichments for muscle and saliva initially expressed as δ^2^H‰ were converted to atom percent excess using standard equations as previously described (Wilkinson et al., 2014).

#### Intramuscular Signaling

Western blot analyses were performed on the sarcoplasmic fraction obtained during myofibrillar isolation [previously described (Smeuninx et al., 2018)]. Sarcoplasmic protein content was determined by a DC protein assay before western blot aliquots of 2 μg protein per 1 μL were prepared in 4× Laemmli sample buffer and sucrose lysis buffer and subsequently boiled for 5 min. Equal amounts of protein (30 μg) were loaded onto 8–12.5% gels and separated by SDS-PAGE for ∼1 h. Following electrophoresis, proteins were transferred onto a BioTrace nitrocellulose or PVDF membrane (Pall Laboratory, Portsmouth, United Kingdom) for 1 h at 100 V. Membranes were subsequently blocked in 2.5–5% skimmed milk for 1 h and washed 3 times for 5 min in TBST before overnight incubation at 4°C in the following primary antibodies (1:1000) in TBST or 2.5% bovine serum albumin (BSA): phospho-70 kDa S6 protein kinase (p70S6K1) Thr389 (#9205), total p70S6K1 (#9202), phospho-eukaryotic initiation factor 4E binding protein (4E-BP1) Thr37/46 (#9459), total 4E-BP1 (#9452), phospho-eukaryotic elongation factor 2 (eEF2) Thr56 (#2331), total eEF2 (#2332), phospho-protein kinase B (Akt) Ser473 (#3787), total Akt (#9272), phospho-AMP activated protein kinase a (AMPKα) Thr172 (#2535), total AMPKα (#5831), phospho-p44/42 MAPK (Erk 1/2) Thr202/Tyr204 (#4370), total p44/42 MAPK (#4695), phospho-S6 Ser240/244 (#5364), total S6 (#2217), phospho-tuberous sclerosis 2 (TSC2) Thr1462 (#3611), total TSC2 (#4308; Cell Signaling Technology, United Kingdom). Membranes were washed three times for 5 min in TBST and incubated for 1 h in their respective secondary antibody [Anti-Rabbit IgG, HRP-linked Antibody (#7074)] (1:10000) and washed again three times for 5 min in TBST. Protein quantification was achieved by incubating the membranes for 5 min in Immobilon Western chemiluminescent HRP substrate (Merck Millipore, Watford, United Kingdom) before being imaged using a G:BOX Chemi XT4 imager using GeneSys capture software (Syngene, Synoptics Ltd., Cambridge, United Kingdom). Bands were quantified using Gene Tools analysis software (SynGene, Synoptics Ltd., Cambridge, United Kingdom).

#### Calculations

The fractional synthetic rate (FSR) of myofibrillar protein was calculated using the standard precursor-product method as described previously (Chinkes et al., 1993). In brief:

FSR(%day-1)=[(EA⁢l⁢a⁢2-EA⁢l⁢a⁢1)EB⁢W⁢x⁢t]x 3.7x 100

Where E_*AlaX*_ is the protein-bound enrichment (in atom percent excess) from muscle biopsies at time X. EBW is the mean ^2^H enrichment (in atom percent excess) in total body water between the time points. Lastly, *t* is the tracer incorporation time in days. Multiplication by 3.7 adjusts for the average number of ^2^H atoms that can become incorporated into alanine, and multiplication by 100 converts the values to percentages.

#### Statistics

Baseline characteristics were compared using an independent samples *t*-test. Muscle protein synthesis and intramuscular signaling were compared using a mixed-design ANOVA, one between-group factor (group) and one within group factor (time). Bonferroni *post hoc* correction was applied to correct for multiple comparisons. Significance was set at *P* < 0.05. Data are presented as means ± standard deviation unless otherwise indicated. All analyses were performed using SPSS version 25 for Windows (SPSS, Inc., Chicago, IL, United States).

## Results

### Participant Characteristics

Participant anthropometric and training characteristics are detailed in Table 1. No significant differences were apparent in any of the baseline characteristics between the groups, with the exception of exercise training background.

### Exercise, Physical Activity, and Dietary Intake

Resistance exercise, physical activity and dietary intake characteristics are presented in Table 2. No significant difference was apparent between OC and MA in bilateral knee extension 1RM strength and total exercise volume completed (the product of sets × repetition × load) was similar between OC and MA. The average Borg CR-10 rating of perceived exertion over the course of the exercise was similar between OC and MA. Average daily step count over the course of the study was not significantly different between OC and MA. Average daily energy and macronutrient intake from the standardized diet was not significantly different between groups.

**TABLE 2:** Exercise characteristics, physical activity and standardized dietary intake during assessment of iMyoPS.

	**OC (*N* = 8)**	**MA (*N* = 7)**	***P*-value**
Bilateral knee extension 1RM (kg)	92 ± 18	100 ± 17	0.311
Total resistance exercise volume (kg)	3861 ± 581	4277 ± 373	0.129
Average borg CR-10 rating	8 ± 2	8 ± 2	0.679
Average daily step-count	8386 ± 1708	7746 ± 1831	0.496
Total energy (kcal⋅day^–1^)	2092 ± 81	2079 ± 101	0.552
Relative carbohydrate (g⋅kg^–1^⋅day^–1^)	4.3 ± 0.3	4.4 ± 0.2	0.603
Relative fat (g⋅kg^–1^⋅day^–1^)	0.7 ± 0.1	0.8 ± 0.1	0.482
Relative protein (g⋅kg^–1^⋅day^–1^)	1.2 ± 0.2	1.3 ± 0.3	0.277

*Data presented as mean ± SD. 1RM, one-repetition maximum; Borg CR-10, borg category ratio (anchored at 10).*

### Myofibrillar Protein Synthesis

**FIGURE 2:**
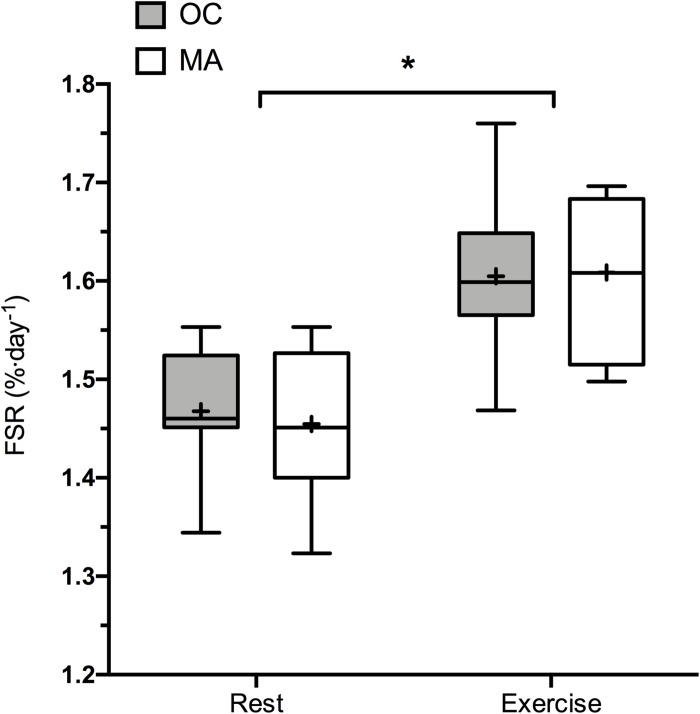
48 h Integrated myofibrillar Protein Synthesis rates in the rested state and following a bout of resistance exercise in older untrained controls (OC, gray bars) and Master Athletes (MA, white bars). The symbol “^∗^” indicates significantly different from rest values (*P* < 0.05). Values are presented as the median (central horizontal line), 25th and 75th percentiles (box), minimum and maximum values (vertical lines) and mean (cross).

**FIGURE 3:**
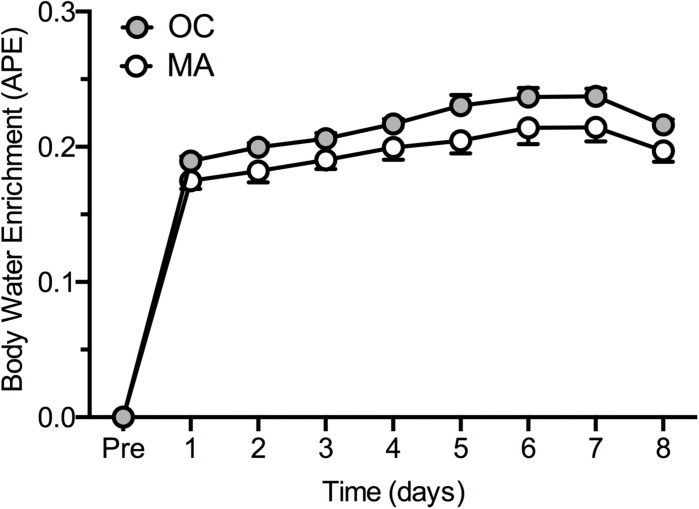
Body water ^2^H enrichment (Atom Percent Excess; APE) in older untrained controls (OC, gray circles) and Master Athletes (MA, white circles). Values are presented as means ± SEM.

Rates of iMyoPS are shown in Figure 2. Rates of iMyoPS were not different between OC (1.47 ± 0.064%⋅day^–1^) and MA (1.45 ± 0.078%⋅day^–1^). iMyoPS increased significantly in response to RE in both OC (1.60 ± 0.083%⋅day^–1^, *P* = 0.032) and MA (1.61 ± 0.078%⋅day^–1^, *P* = 0.034), with no significant difference between groups. Body water ^2^H enrichment, assessed via saliva samples, increased significantly above rested-state values at 24 h after consumption of the first dose (MA: 0.19 ± 0.01 APE, OC: 0.17 ± 0.02 APE; *P* < 0.001 for both). Steady-state isotopic enrichment was maintained throughout the remainder of the study, with no significant difference between groups at any time point (Figure 3).

### Intramuscular Anabolic Signaling

**FIGURE 4:**
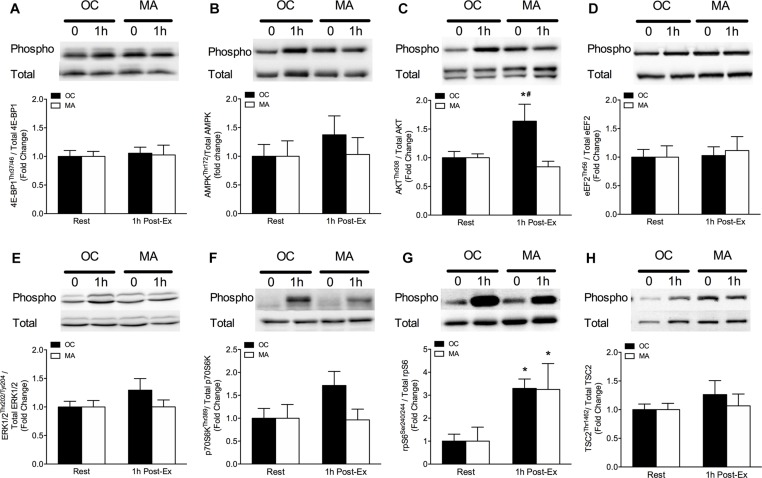
Intramuscular anabolic signaling of **(A)** 4E-BP1^*Thr37*/46^, **(B)** AMPK^*Thr72*^, **(C)** Akt^*Thr308*^, **(D)** eEF2^*Thr56*^, **(E)** ERK1/2^*Thr202*/*Tyr204*^, **(F)** p70S6K^*Thr389*^, **(G)** rpS6^*Ser240*/244^, and **(H)** TSC2^*Thr1462*^ at rest and 1 h post-resistance exercise in older untrained controls (OC, black bars) and Master Athletes (MA, white bars). All proteins are expressed relative to their respective total protein abundance. Significance was set at *P* < 0.05. The symbol “^∗^” indicates significantly different from rest (*P* < 0.05). The symbol “#” indicates significantly different from MA (*P* < 0.05). Values are presented as means ± SEM.

Intramuscular anabolic signaling markers are shown in Figure 4. Phosphorylation of Akt^*Thr308*^ increased significantly from rest in OC only at 1 h post-exercise (*P* = 0.032) and phosphorylation of Akt^*Thr308*^ was significantly greater in OC compared with MA at 1 h post-exercise (*P* < 0.05). Phosphorylation of p70S6K^*Thr241*^ tended to increase above rest in OC at 1 h post-exercise (*P* = 0.064) with a trend for a greater phosphorylation in OC compared with MA at 1 h post-exercise (*P* = 0.079). Phosphorylation of RPS6^*Ser240/244*^ increased significantly from rest in OC (*P* = 0.033) and MA (*P* = 0.048) at 1 h post-exercise, with no difference between groups.

## Discussion

The loss of skeletal muscle mass is a commonly observed consequence of aging (sarcopenia). Age-related muscle anabolic resistance is touted as a key mechanism contributing to the development and progression of sarcopenia (Shad et al., 2016) and is thought to be exacerbated by aspects of biological aging [i.e., inactivity, obesity (Smeuninx et al., 2017)]. Individuals that have undertaken regular structured exercise training throughout a large proportion of adulthood (MA), typically display superior physiological function and indices of muscle morphology compared with age-matched non-athletes (OC) (Zampieri et al., 2015; McKendry et al., 2018, 2019) and provide an opportunity to unpick the role of inherent and biological aging processes on muscle mass regulation (Lazarus and Harridge, 2007). However, there is a dearth of *in vivo* mechanistic information of muscle metabolic regulation in MA (McKendry et al., 2018). Herein, we provide the first comparison of rested-state and exercise-induced iMyoPS rates between endurance-trained MA and OC. Our findings demonstrate no discernible difference in rested-state nor, contrary to our hypothesis, exercise-induced iMyoPS rates between MA and OC. Furthermore, we observed no clear difference in the mTORC1-mediated signaling response to exercise between MA and OC. Taken together, these data suggest that despite divergent long-term exercise habits in MA, OC possess a similar capacity to upregulate intramuscular signaling and iMyoPS in response to unaccustomed exercise contraction.

Understanding the influence of long-term high-level exercise training on mechanisms of skeletal muscle aging is of great importance in the development of interventions to delay or reverse sarcopenia progression. Generally, physical activity declines with advancing age (Jefferis et al., 2014) and inactivity and obesity may accelerate the development of muscle anabolic resistance and sarcopenia (Breen et al., 2013; Steffl et al., 2017). In contrast, MA, who have remained highly active through a large portion of adulthood maintain a healthy body composition and typically display superior physiological function and indices of muscle morphology compared with OC (Zampieri et al., 2015; McKendry et al., 2018, 2019). Nonetheless, it was recently demonstrated that middle-aged master triathletes aged ∼53 years display a blunted iMyoPS response to a downhill running stimulus compared with young triathletes aged ∼27 years (Doering et al., 2016). The absence of a rested-state iMyoPS measurement and untrained age-matched cohort in this study, precluded the authors from identifying how training status influences the exercise-induced iMyoPS response with aging; shortcomings that we have attempted to address herein.

Given evidence that exercise-induced iMyoPS response may diminish with advancing age (Brook et al., 2016) and that chronic exercise training may alter rested and exercise-induced MPS responses (Short et al., 2004; Wilkinson et al., 2008) we expected that our MA cohort, with an average of ∼50 years of consistent training habits, would demonstrate divergence in exercise-induced iMyoPS compared with OC. Surprisingly, whilst rested-state iMyoPS rates were similar between MA and OC and comparable to values reported elsewhere in older individuals (∼1.4–1.5%⋅day^–1^) (McGlory et al., 2018; Bell et al., 2019), the capacity to increase iMyoPS rates in response to unaccustomed RE did not differ between MA and OC (∼10% increase over 48 h post-exercise). The absence of any difference in iMyoPS between groups may be due to the relatively similar characteristics of MA and OC. In our recent work in MA and OC cohorts similar to those in the present study (albeit different volunteers), we demonstrated lower fat mass and greater skeletal muscle index in MA vs. OC (McKendry et al., 2019). In contrast, anthropometric characteristics were indistinguishable between groups in the present study, which was likely compounded by the relatively small sample size. Our choice not to replicate the array of assessments from our previous work (to reduce the burden on participants) means we are unable to present any physiological or morphological differences between the current MA and OC cohorts. Nonetheless, our findings demonstrate that long-term highly active MA, habitually completing ∼4–5 sessions of structured endurance exercise training a week (totaling ∼8 h), do not display a greater capacity to upregulate iMyoPS with an unaccustomed exercise stimulus compared with healthy OC with no history of endurance exercise training.

Previous studies utilizing acute intravenous infusions of stable isotope tracers have demonstrated age-related muscle anabolic resistance in response to exercise (Kumar et al., 2009) and protein feeding (Moore et al., 2015; Wall et al., 2015; Smeuninx et al., 2017). The incorporation of an orally ingested D_2_O isotope tracer in the present study, enabled us to assess rested-state and exercise-induced iMyoPS in free-living conditions over 48 h, unconstrained by a strictly controlled laboratory environment. Given recent evidence that iMyoPS rates measured via D_2_O are associated with muscle mass accretion in younger individuals (Damas et al., 2016), our findings may be indicative of the potential for skeletal muscle remodeling over a prolonged period in our cohorts. Specifically, the capacity for exercise-induced muscle remodeling following an unaccustomed RE stimulus appears to be similar between MA and OC. Important to note, is that the muscle protein breakdown response to exercise contraction may also play an important role in skeletal muscle remodeling (Tipton et al., 2018). Therefore, it cannot be discounted that disparities exist in the rates of proteolysis between OC and MA, with important implications for muscle morphology.

Our choice to incorporate an unaccustomed RE stimulus in an endurance-trained MA cohort for comparison with age-matched untrained OC, warrants clarification. RE is, to date, the most effective non-pharmacological stimulus for the synthesis of myofibrillar protein, the critical component of muscle contractile mass. However, RE-induced myofibrillar protein remodeling appears to be impaired in older compared with younger individuals (Kumar et al., 2009; Brook et al., 2016; Phillips et al., 2017). Given the proposed link between physical activity and muscle anabolic responsiveness in older age, our primary aim was, therefore, to understand whether individuals who had undertaken long-term high-level exercise displayed greater RE-induced myofibrillar protein remodeling compared with OC. The RE stimulus used here was likely equally unaccustomed to both MA and OC, which is supported by the observation that a substantial degree of exercise-induced muscle plasticity is conserved in endurance trained athletes undertaking acute RE (Coffey et al., 2006). In contrast, repeated exposure to aerobic exercise (AE) leads to a phenotype-specific increase in mitochondrial protein synthesis rates in endurance-trained young and old individuals (Wilkinson et al., 2008; Robinson et al., 2017). Thus, incorporating an AE stimulus in the current study may have resulted in a divergent myofibrillar protein remodeling response between groups. Our findings demonstrate a similar level of RE load-volume and perceived effort between MA and OC, with no effect of long-term exercise training on the capacity to upregulate iMyoPS of myofibrillar protein in MA. Fraction-specific iMyoPS responses to divergent modes of exercise in endurance- and strength-trained MA warrants further investigation to further unravel how long-term training modulates muscle adaptive remodeling.

Age-related blunting of exercise-induced muscle anabolism may be underpinned by impairments in translational efficiency (Kumar et al., 2009; Brook et al., 2016). To gain further insight into the mechanistic regulation of iMyoPS in MA and OC, rested-state and exercise-induced intramuscular signaling intermediates were measured. At 1 h post-exercise, rpS6 phosphorylation increased above rest in OC and MA, whereas the phosphorylation of Akt increased and p70S6K tended to increase above rest in OC only. It has been suggested that phosphorylation of mTORC1-mediated signaling events with exercise is delayed with aging, and it is possible that a peak response may have occurred >1 h post-exercise in our participants (Drummond et al., 2008; Farnfield et al., 2012; Smeuninx et al., 2018). Thus, inclusion of additional biopsy collection points over the first several hours of exercise recovery may provide greater insight of the anabolic signaling regulation in MA and OC. We did not analyze mTORC1-mediated signaling in the 48 h post-exercise biopsy, as these events typically subside well before this point. Another possible explanation for the absence of significant exercise-induced stimulation in mTORC1-mediated signaling, or potential differences between MA and OC, may be related to the fasted-state biopsy sampling, which was chosen to isolate an exercise-only effect. Given that role of protein provision is maximizing intramuscular anabolic signaling and MPS, it would be prudent to investigate how regulatory signaling intermediates respond to these combined anabolic stimuli in MA and OC. Taken together, rested and exercise-induced mTORC1-mediated signaling was generally indistinguishable between MA and OC, mirroring the equivalent iMyoPS rates between groups.

## Conclusion

In conclusion, we have demonstrated equivalent iMyoPS rates and an intracellular signaling profile in OC and MA, in the rested-state and in response to a novel RE stimulus. The superior physiological function (VO_2max_) and muscle morphology (Type I fiber shift, fiber capillarization, etc.) reported elsewhere in endurance-trained MA compared with OC can be explained by repeated exposure to AE stimuli over an extended period. Nonetheless, despite a long-term history of endurance training in MA, the capacity for myofibrillar protein remodeling in response to unaccustomed RE appears to be similar to that in healthy untrained OC.

## Data Availability

The datasets generated for this study are available on request to the corresponding author.

## Ethics Statement

Ethical approval was obtained through the East Midlands – Derby Research Ethics Committee (18/EM/0004) and conformed to the requirements of Research Governance at the University of Birmingham Research Governance, as the study sponsor.

## Author Contributions

All authors gave their final approval of the version of the manuscript to be published. JM, CG, SP, and LB designed the study. JM, BJS, GW, and LB organized and carried out the experiments with the assistance of BS. JM, BJS, BS, SO, CG, SP, and LB performed the data analyses. JM and LB performed the statistical analysis of the data. JM, BJS, CG, SP, and LB wrote the manuscript. JM, SP, and LB were the guarantors of this work and took responsibility for the integrity and accuracy of the data analysis.

## Conflict of Interest Statement

The authors declare that the research was conducted in the absence of any commercial or financial relationships that could be construed as a potential conflict of interest.

## Footnotes

1 https://www.ons.gov.uk
